# Biophysical Insights into How Surfaces, Including Lipid Membranes, Modulate Protein Aggregation Related to Neurodegeneration

**DOI:** 10.3389/fneur.2013.00017

**Published:** 2013-03-01

**Authors:** Kathleen A. Burke, Elizabeth A. Yates, Justin Legleiter

**Affiliations:** ^1^C. Eugene Bennett Department of Chemistry, West Virginia UniversityMorgantown, WV, USA; ^2^NanoSAFE, West Virginia UniversityMorgantown, WV, USA; ^3^The Center for Neurosciences, West Virginia UniversityMorgantown, WV, USA

**Keywords:** amyloid disease, lipid membranes, protein aggregation, Alzheimer’s disease, Huntington’s disease, Parkinson’s disease, prion disease

## Abstract

There are a vast number of neurodegenerative diseases, including Alzheimer’s disease (AD), Parkinson’s disease (PD), and Huntington’s disease (HD), associated with the rearrangement of specific proteins to non-native conformations that promotes aggregation and deposition within tissues and/or cellular compartments. These diseases are commonly classified as protein-misfolding or amyloid diseases. The interaction of these proteins with liquid/surface interfaces is a fundamental phenomenon with potential implications for protein-misfolding diseases. Kinetic and thermodynamic studies indicate that significant conformational changes can be induced in proteins encountering surfaces, which can play a critical role in nucleating aggregate formation or stabilizing specific aggregation states. Surfaces of particular interest in neurodegenerative diseases are cellular and subcellular membranes that are predominately comprised of lipid components. The two-dimensional liquid environments provided by lipid bilayers can profoundly alter protein structure and dynamics by both specific and non-specific interactions. Importantly for misfolding diseases, these bilayer properties can not only modulate protein conformation, but also exert influence on aggregation state. A detailed understanding of the influence of (sub)cellular surfaces in driving protein aggregation and/or stabilizing specific aggregate forms could provide new insights into toxic mechanisms associated with these diseases. Here, we review the influence of surfaces in driving and stabilizing protein aggregation with a specific emphasis on lipid membranes.

## Introduction

**Figure 1:**
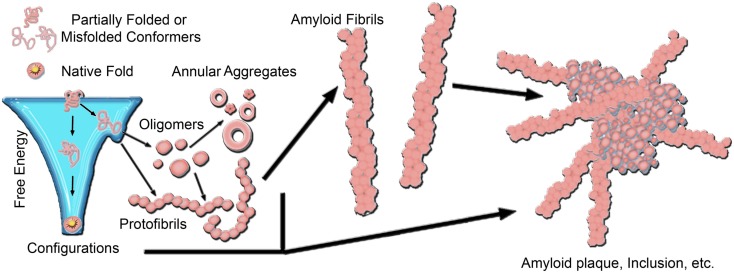
**A generic aggregation scheme for amyloid-forming proteins**. Proteins fold into their native structure, which is typically a low free energy configuration. However, the energy landscape for protein folding often can have localized minima in which a protein can become trapped into a misfolded conformation, which can lead to aggregation into β-sheet rich amyloid fibrils. The formation of fibrils often proceeds through a heterogeneous mixture of intermediate species, including oligmers and protofibrils. Off-pathway aggregates can also form, such as annular aggregates. These aggregates accumulate into amyloid plaques or inclusions in the diseased brain. The aggregation pathway for any given amyloid-forming protein can vary considerably depending on the protein and its folding environment.

A common motif of several neurodegenerative diseases is the ordered aggregation of specific proteins, leading to their deposition in tissues or cellular compartments (Chiti and Dobson, 2006). Often referred to as protein conformational or misfolding disorders, such diseases include Alzheimer’s disease (AD), Parkinson’s disease (PD), Huntington’s disease (HD), amyloidoses, α1-antitrypsin deficiency, and the prion encephalopathies to name a few. The common structural motif of protein aggregates associated with these diseases is the formation of extended, β-sheet rich fibrils, referred to as amyloid. Despite no apparent correlation between aggregating proteins in size or primary amino acid sequence, the characteristic lesions of each disease typically contain fibrillar structures with common biochemical characteristics (Dobson, 2003; Chiti and Dobson, 2006), indicating the potential for a conserved mechanism of pathogenesis linking these phenotypically diverse diseases. The earliest potential event in the disease process may be the conversion of a protein to a critical abnormal conformation, resulting in toxic gain of function for the monomer, and/or the formation of toxic nanoscale aggregates (Figure 1; Naeem and Fazili, 2011). The elusive toxic species, whether monomeric or higher-order, may subsequently initiate a cascade of pathogenic protein–protein interactions that culminate in neuronal dysfunction. The precise timing of such interactions and the mechanisms by which altered protein conformations or aggregates trigger neuronal dysfunction are unclear.

The formation of fibrils often proceeds via a heterogeneous mixture of intermediate aggregate structures, including a variety of protofibrils and oligomers (Figure 1). Amyloid formation typically occurs via a nucleation-growth mechanisms that features an initial lag-phase due to a thermodynamically unfavorable nucleation event (Lomakin et al., 1996; Murphy, 2002; Chiti and Dobson, 2006). Once nucleation occurs, aggregation proceeds via an exponential growth phase associated with the addition of monomers into aggregate forms. The initial lag-phase can be circumvented by the presence of pre-existing aggregates that can act as seeds for amyloid formation (Lansbury, 1997; Hu et al., 2009; Langer et al., 2011; Hamaguchi et al., 2012). To further complicate the issue, several aggregates have been identified that may be off-pathway to fibril formation, such as annular structures (Wetzel, 1994; Wacker et al., 2004). While there are often specific mutations or dysfunctional processing that can be directly linked to aggregation, the nature, and location of protein aggregates *in vivo* depends on the specific protein associated with disease. The specific protein involved also influences the specific form of the critical aggregation nucleus. For example, synthetic polyglutamine (polyQ) peptides are thought to have a monomeric critical nucleus (Chen et al., 2002a,b; Wetzel, 2012); however the addition of flanking sequences associated with the first exon of the huntingtin (htt) protein can change the size of the critical nucleus to a tetramer (Jayaraman et al., 2012; Wetzel, 2012). This can be further modulated by the addition of β-hairpin motifs within the polyQ domain (Kar et al., 2013). The extent of the lag-phase, and subsequent aggregation of polyQ peptides and htt proteins is dependent on the size of the polyQ domain (Legleiter et al., 2010; Kar et al., 2011). As protein aggregation often progresses from misfolded monomers to oligomeric precursors and finally mature fibrils, intensive research activity has been devoted to determining the most toxically relevant aggregate species in many of these diseases. This is particularly important, as for the vast majority of these diseases, there are no widely effective preventative measures or therapeutic treatments.

Fibril structures associated with several different amyloid-forming proteins have been experimentally resolved, and a common motif of fibrillar aggregates is a cross-β structure (Eanes and Glenner, 1968; Glenner et al., 1971; Kirschner et al., 1986; Sunde et al., 1997; Berriman et al., 2003; Tycko and Ishii, 2003; Tycko, 2004, 2006; Nelson et al., 2005; Fandrich, 2007). While the structural spine of fibrils share this common intermolecular β-sheet structure, a variety of possibilities are available for the packing of protofilaments into the fibril structure, even for the same protein/peptide. This variability can lead to distinct amyloid fibril morphologies. Such variable protofilament arrangements give rise to distinct fibril morphologies, often termed polymorphisms (Kodali and Wetzel, 2007). For example, Aβ has been shown to form a variety of fibril structures *in vitro* dependent on the peptide preparation and aggregation conditions (Kodali et al., 2010). Furthermore, fibril polymorphs have been observed for several other amyloid-forming proteins, such as calcitonin (Bauer et al., 1995), amylin (Goldsbury et al., 1997), glucagon (Pedersen et al., 2006), the SH3 domain of phosphatidylinositol-3′-kinase (Jimenez et al., 1999; Chamberlain et al., 2000; Pedersen et al., 2006), insulin (Bouchard et al., 2000; Jimenez et al., 2002; Dzwolak et al., 2004), and lysozyme (Chamberlain et al., 2000). Polymorphic fibrils can differ in the cross-sectional thickness or helical pitch of the fibril, which can be observed via high resolution imaging techniques like transmission electron microscopy (TEM) and atomic force microscopy (AFM) or distinguished with spectroscopic techniques like circular dichroism (CD; Petkova et al., 2005; Kurouski et al., 2010, 2012; Mossuto et al., 2010; Norlin et al., 2012). While polymorphs are often observed for various *in vitro* aggregation reactions, polymorphs have been observed in amyloid fibrils extracted from tissue as well (Crowther and Goedert, 2000; Jimenez et al., 2001), affirming that *in vivo* aggregation can be heterogeneous and complex. Furthermore, it has been proposed that polymorphic fibrils may result in distinct biological activities and variable toxicity related to the different aggregate structures (Seilheimer et al., 1997; Petkova et al., 2005). These distinct fibril morphologies may also have distinct aggregate intermediates associated with their formation, adding to the heterogeneity of potential protein aggregates and further complicating efforts aimed at elucidating the relative role of discrete aggregates in disease-related toxicity.

While protein preparation and environment influence the structural polymorphs of protein aggregates *in vitro*, determining what environmental factors influence aggregation *in vivo* remains difficult. However, the interaction of proteins at solid interfaces, including cellular membranes comprised of lipid bilayers, may prove to be a fundamental phenomenon with potential implications for protein-misfolding diseases. Solid surfaces, such as mica, graphite, gold, and Teflon, have been shown to heavily influence aggregation kinetics and the resulting aggregate morphology for a variety of amyloid-forming proteins (Goldsbury et al., 1999; Hoyer et al., 2004; Morriss-Andrews and Shea, 2012). A variety of kinetic and thermodynamic studies point to significant conformational changes being induced in proteins encountering surfaces (Gray, 2004). These surface induced conformational changes in proteins could play a critical role in nucleating amyloid formation or altering aggregate morphology to specific toxic species. Such phenomenon are well demonstrated by a study of immunoglobulin light-chain aggregation on mica (Zhu et al., 2002). Small pieces of mica were incubated in solutions containing a recombinant amyloidogenic light-chain variable domain of smooth muscle actin (SMA) antibody, under conditions in which fibrils normally do not form (i.e., low concentration and no agitation). At short times, amorphous aggregates appeared on mica, and fibrils were observed within 10 h and fibrils were not formed in the solution within the same time frame. The fibrils on the surface of mica grew from the amorphous aggregates and the assemblies of oligomers present on mica. The use of such solid surfaces as model systems provides the opportunity to elucidate how specific surface environment influence protein aggregation.

In regards to disease-related protein aggregation, surfaces of more physiological relevance are cellular and subcellular membranes that are predominately comprised of lipid bilayers. Like solid surfaces, the presence of lipid membranes can alter the aggregation of disease-related proteins by increasing aggregation rates, nucleating aggregation, promoting specific polymorphs, or even stabilizing potentially toxic, transient aggregate intermediates. A significant question remains regarding why amyloid fibrils form *in vivo* at concentrations that are orders of magnitude lower (Seubert et al., 1992) than the critical nucleation concentrations required *in vitro* (Lomakin et al., 1996; Sabate and Estelrich, 2005). A possible answer is the ability to create local concentrations of protein adsorbed onto molecular surfaces, such as cellular and subcellular membranes (Kim et al., 2006; Aisenbrey et al., 2008). Lipid interaction appears to be a common modulator in fibril formation, as studies of α-synuclein (α-syn; Jo et al., 2000, 2004; Necula et al., 2003), islet amyloid polypeptide (IAPP; Knight and Miranker, 2004), and β-amyloid (Aβ; McLaurin and Chakrabartty, 1996, 1997; Choo-Smith et al., 1997; Yip and McLaurin, 2001; Yip et al., 2002) all demonstrate accelerated fibril formation in a membrane environment in comparison to bulk solution. General physicochemical properties of lipid membrane, including phase state, bilayer curvature, elasticity, and modulus, surface charge, and degree of hydration, modulate protein aggregation (Gorbenko and Kinnunen, 2006). The exact chemical composition and lipid constituents of a lipid bilayer can also influence the aggregation process (Evangelisti et al., 2012). Potentially important chemical properties of membrane components include the extent of acyl chain unsaturation, conformation and dynamics of lipid headgroups and acyl chains, and protein–lipid selectivity arising from factors such as the hydrophobic matching at the protein–lipid interface (Jensen and Mouritsen, 2004). Although lipid bilayers may act catalytically to induce aggregation by providing environments that promote protein conformation and orientation conducive to fibril assembly (Thirumalai et al., 2003; Sparr et al., 2004; Zhao et al., 2004), cell membranes may also be targeted by protein aggregates to induce physical changes in the membrane, leading to dysfunction and cell death. This may be due to the ability of amyloid-forming peptides to induce membrane permeabilization by altering bilayer structure via the sequestration of membrane components into fibrils (Michikawa et al., 2001; Lins et al., 2002; Sparr et al., 2004; Zhao et al., 2004; Valincius et al., 2008) or by forming unregulated pore-like structures (Jang et al., 2007). A variety of amyloid-forming proteins, including Aβ, IAPP, and htt, have been show to locally change the rigidity of model lipid bilayers in a generic manner (Burke et al., 2013). Furthermore, the presence of lipid membranes can also influence the ability of small molecules to prevent or destabilize protein aggregates, having a major impact on several therapeutic strategies. Such a scenario has been demonstrated experimentally as (-)-epigallocatechin gallate (EGCG), which has been shown to inhibit the aggregation of several amyloid-forming proteins in the absence of surfaces (Bieschke et al., 2010; Popovych et al., 2012), was less effective at inhibiting aggregation of human IAPP at a phospholipid interface (Engel et al., 2012).

Here, we review the influence of surfaces in driving and stabilizing protein aggregation with a specific emphasis on lipid membranes. We will initially focus on Aβ as an illustrative example, and then quickly review some interesting features of the interaction of other select amyloid-forming proteins with surface interfaces.

## The Aggregation of Aβ on Solid Surfaces

The ordered aggregation of Aβ into neuritic plaques is one of the major hallmarks of AD. Aβ is a secreted peptide derived from the endoproteolysis of the amyloid precursor protein (APP), a receptor-like transmembrane protein, and is ubiquitously expressed in neural and non-neural cells. Successive cleavage of APP by β-secretase and γ-secretase results in the release of an intact Aβ peptide. Aβ contains a portion of APP’s transmembrane domain, as well as an extracellular portion, resulting in an amphiphilic peptide ∼39–43 residues in length. The Aβ component of amyloid plaques found in the diseased brain consist primarily of two versions of the peptide, which are 40 and 42 amino acids long [Aβ(1–40) and Aβ(1–42) respectively]. Aβ(1–42) aggregates more quickly than Aβ(1–40) and is thought to play a major role in AD (Jarrett and Lansbury, 1993). The amphiphilic nature of Aβ is thought to drive its aggregation and may play an important role in its interaction with solid surfaces and ability to insert and/or penetrate lipid membranes (Lansbury and Lashuel, 2006; Williams and Serpell, 2011). The extra addition of two hydrophobic residues in Aβ(1–42) may also lead to variations in the interaction of this peptide with surfaces compared to Aβ(1–40).

Hydrophobic Teflon surfaces can be considered mimics of the non-polar plane of lipid membranes. While both Teflon and Aβ carry a negative charge at physiological pH, protein dehydration effects lead to substantial adsorption of Aβ at pH 7(Giacomelli and Norde, 2003). Aβ(1–40) and Aβ(1–42) adsorption to Teflon particles increased aggregation and fibrillogenesis (Linse et al., 2007). Adsorption of Aβ to another hydrophobic surface, highly ordered pyrolytic graphite, results in extended aggregate formation in a nucleation dependent manner (Kowalewski and Holtzman, 1999). Using a variety of surfaces with tunable hydrophobicity or hydrophilicity (as well as supported lipid bilayers), weakly adsorbed peptides with two-dimensional diffusivity were found to be critical precursors to surface growth of Aβ(1–42) fibrils (Shen et al., 2012). As the adsorption of Aβ on highly hydrophilic surfaces was negligible, fibril growth was inhibited on such surfaces. On highly hydrophobic surfaces, the two-dimensional diffusion of Aβ along the surface was too low, also inhibiting fibril formation. It appears that surface properties that promote weak adsorption of Aβ to the surface and maintain translational mobility result in local concentrations of Aβ due to confinement within the plane of the surface, allowing for fibril formation at a concentration far below the critical concentration observed in bulk solutions. The adsorption of Aβ to hydrophilic silica surfaces is pH dependent, occurring at pH 4 and 7 when Aβ has an overall positive charge (Giacomelli and Norde, 2005), suggesting a vital role of electrostatics on Aβ‘s adsorption to surfaces.

**Figure 2:**
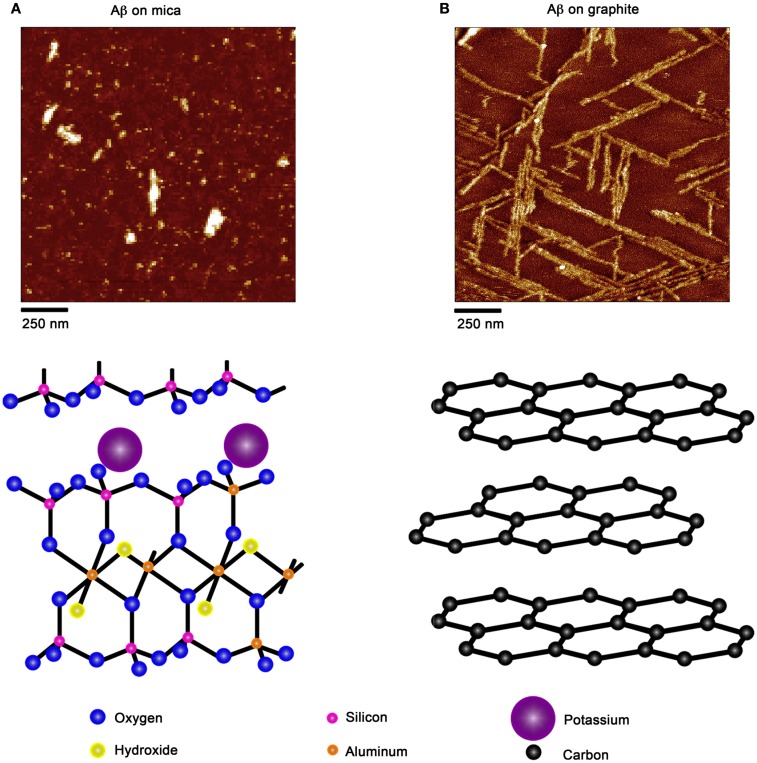
**Aβ aggregation is modulated by the presence of chemically distinct solid surfaces**. **(A)** On highly ordered pyrolytic graphite, Aβ aggregates into extended nanoribbons that are epitaxially ordered on the surface. The distinct orientation of Aβ aggregates on graphite is attributed to the optimization of the contact between the peptide and underlying hydrophobic carbon lattice. **(B)** On a negatively charged, hydrophilic mica surface, Aβ forms discrete oligomers that maintained some lateral mobility along the plane of the surface. These oligomers could organize into elongated protofibrillar structures. Schematic representations of the structure of each surface (graphite and mica) are provided under each image.

**Figure 3:**
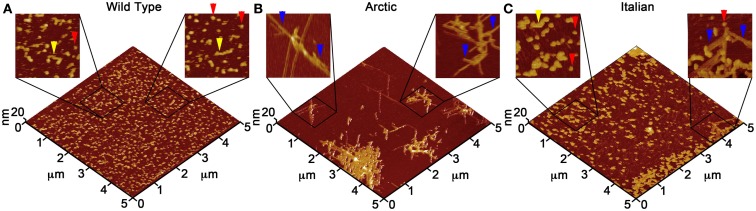
**Point mutations in Aβ(1–40) modulate aggregate morphology in the presence of a mica surface**. Using solution AFM, the aggregation of Wild Type, Arctic (E22G), and Italian (E22K) Aβ was monitored on a mica surface (Aβ concentration was 20 μM for all experiments). 5 μm × 5 μm images are presented in 3D with indicated zoomed in areas of 1 μm × 1 μm shown in 2D. **(A)** Wild Type Aβ formed a large population of oligomers (red arrows) and highly curved, elongated protofibrils (yellow arrows) with aggregate heights of ∼3–5 nm similar to presented in Figure 2. **(B)** Arctic Aβ formed rigid, branched, and highly ordered fibrillar aggregates (blue arrows) along the crystallographic lattice of mica with aggregate heights of ∼2–5 nm along the contour. These Arctic Aβ aggregates morphologically distinct from those formed by Wild Type Aβ. **(C)** Italian Aβ predominately aggregated into small oligomers (2–3 nm tall, red arrows) that coalesced into larger protofibrils (yellow arrows), in a similar fashion to Wild Type Aβ; however, a small number of rigid, elongated “Arctic-like” fibrillar aggregates of Italian Aβ also formed (blue arrow).

Due to the ability of AFM to be operated in solution and track the formation and fate of individual aggregates with time on surfaces (Goldsbury et al., 1999), the impact of surface chemistry on the morphology of Aβ aggregates has been extensively studied with this technique. On mica, a hydrophilic surface, Aβ(1–40) (Blackley et al., 2000) and Aβ(1–42) (Kowalewski and Holtzman, 1999) form small, highly mobile oligomeric aggregates that organize into extended pre-fibrillar aggregates that continually elongate with time (Figure 2A). These aggregate structures are similar in morphology to those formed in bulk solution from similarly prepped Aβ stocks (Kowalewski and Holtzman, 1999; Legleiter and Kowalewski, 2004). However, Aβ(1–42) aggregates into morphologically distinct structures on a graphite surface (Figure 2B), forming extended nanoribbons with heights of ∼1–1.2 nm and widths of ∼18 nm (Kowalewski and Holtzman, 1999). These dimensions suggest that Aβ adopts a fully extended β-sheet conformation perpendicular to the long axis of the nanoribbons. These nanoribbons elongated with time, organize themselves into parallel, raft-like structures with a preferential alignment along the graphite lattice. Aβ adsorbs to and aggregates on surfaces functionalized with methyl, carboxyl, or amine groups; however, aggregate morphology and surface affinity is dependent on the specific surface chemistries (Moores et al., 2011). Hydrophobic surfaces promote formation of spherical amorphous clusters; charged surfaces promote the formation of protofibrils (Moores et al., 2011). Studies of the aggregation of Aβ peptides containing single point mutations on mica further support the notion that electrostatics play an important role in Aβ adsorption and aggregation on surfaces (Yates et al., 2011). These mutations are clustered around the central hydrophobic core of Aβ (E22G Arctic mutation, E22K Italian mutation, D23N Iowa mutation, and A21G Flemish mutation) and are associated with familial forms of AD. In bulk solution and under identical preparatory conditions, these Aβ mutants form aggregated species that were morphologically similar to those of Wild Type Aβ; however, on a mica surface the aggregates differ in morphology (Figure 3). While Wild Type Aβ forms oligomers and putative protofibrils on mica similar to other previously described studies, Arctic Aβ aggregate into extended, fibrillar aggregates on mica that orient on the surface similar to the previously described Wild Type Aβ aggregates on graphite. However, the dimensions of the Arctic Aβ aggregates on mica indicate they most likely contain a β-turn as opposed to the fully elongated Wild Type Aβ nanoribbons on graphite. Italian Aβ, which replaces a negatively charged residue with a positive one, adsorbs quickly to mica and predominantly forms oligomeric aggregates reminiscent of those formed by wild type Aβ on mica. However, there was a small percentage of Italian Aβ aggregates similar in morphology to those formed by Arctic Aβ on mica.

## The Interaction of Aβ with Lipid Surfaces

While studies on model surfaces can provide mechanistic detail on how solid interfaces alter and/or promote Aβ aggregation, ultimately, pathological protein aggregation occurs in a cellular environment, dictating the need to study protein-misfolding and aggregation on more physiologically relevant surfaces. This is not to say that studies on solid surfaces are irrelevant. For example, the aforementioned dependence on lateral mobility of Aβ on a surface being critical in fibril formation was directly extended to lipid surfaces as well (Shen et al., 2012). This phenomenon could be important in light of single molecule studies demonstrating that Aβ inserted into anionic lipid membranes demonstrate high lateral mobility until aggregating into oligomers (King et al., 2012).

**Figure 4:**
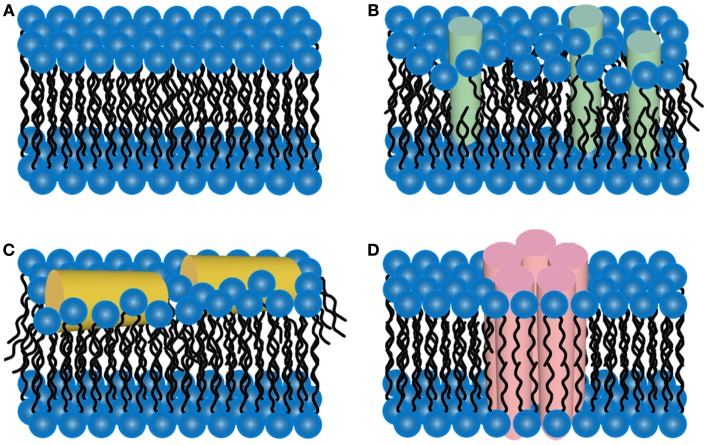
**Schematic representations of potential mechanisms of amyloid/lipid association**. **(A)** A schematic representation of simplified, undisrupted bilayer is presented. This bilayer structure can be perturbed by **(B)** amyloid-protein insertion or **(C)** association of amphiphilic α-helices lipid-binding domains. Such scenarios could lead to membrane thinning and non-specific membrane leakage. **(D)** Many amyloid-forming proteins have been shown to form pore-like structures that can act as unregulated ion-selective channels.

It has been hypothesized that a potential pathway for Aβ toxicity may lie in its ability to modulate lipid membrane function. This hypothesis is based on the observation that Aβ bears a portion of the APP transmembrane domain. Thus, elucidating the interaction between Aβ and membrane lipids could be critical in understanding potential pathways of Aβ toxicity, especially given the results of studies that demonstrate that changes in membrane composition occur in AD along with the association with plaques, tangles, and neuritic dystrophy. Importantly, it has often been observed that exogenously added Aβ will selectively bind a subset of cells in an apparent homogenous population of cells in culture (Lacor et al., 2004; De Felice et al., 2008). Such an initial cellular binding event may play a critical role in toxic mechanisms and cell to cell propagation of disease. This cell selectivity may be influenced by the presence of specific lipid components or membrane properties (Okada et al., 2007; Wakabayashi and Matsuzaki, 2007; Lin et al., 2008). Once Aβ aggregation begins in or near a membrane, the potential toxic mechanism include disruption of the bilayer structure, changes in bilayer curvature, and/or the creation of membrane pores or channels (Arispe et al., 1993a,b; McLaurin and Chakrabartty, 1996, 1997; Mirzabekov et al., 1996; Gorbenko and Kinnunen, 2006; Figure 4). The majority of studies on membrane-mediated fibrillogenesis have been undertaken with model systems including amyloidogenic peptides or proteins and lipid vesicles or supported bilayers of varying composition (Terzi et al., 1997; Lindstrom et al., 2002; Bokvist et al., 2004; Sparr et al., 2004). These studies often point to the importance of the chemical nature of membrane lipids and the mode of protein–lipid interactions in determining fibrillogenic properties of membrane bound Aβ. Lipids can also stabilize toxic protofibrils and even revert mature fibrils into such toxic species (Martins et al., 2008), providing another potential role for lipid surfaces in toxicity.

A large number of biophysical techniques have been applied in understanding the specific interactions between lipid membranes and Aβ. Due to the ability to control bilayer composition, biomimetic unilamellar vesicles have been extensively used to elucidate the interaction between Aβ and membranes (Williams et al., 2010). Simple vesicles comprised of a single lipid component, soybean PC, have been used to demonstrate that the presence of neutral PC delays the characteristic lag time to initiate Aβ aggregation in a lipid concentration-dependent manner (Sabate et al., 2005). Lipids can also induce changes in the secondary structure of Aβ, as CD studies demonstrated that a variety of lipids induce a transition from an α-helical to β-sheet structure in Aβ(McLaurin and Chakrabartty, 1997). As with solid surfaces, the charge of the lipid membrane surfaces, determined by the headgroups of phospholipids, dictate the extent of Aβ/membrane association due to electrostatic considerations. For example, similarly prepared Aβ(1-40) displays a stronger affinity to liposomes comprised of POPG compared to those comprised of POPC, with only the association with POPG enhancing the rate of Aβ aggregation (Kremer and Murphy, 2003). Freshly prepared Aβ(1–40) preferentially binds negatively charged PG membranes and composite membranes containing negatively charged lipids in comparison to neutral membranes; however, the relative affinity for fibril aggregates of Aβ with these lipid membranes is altered (Lin et al., 2007). Allowing Aβ(1–40) to form fibrils causes the affinity for negatively charged membranes to be smaller compared to the affinity for neutral membranes, suggesting that Aβ aggregation state can further modulate the interaction with lipid surfaces.

**Figure 5:**
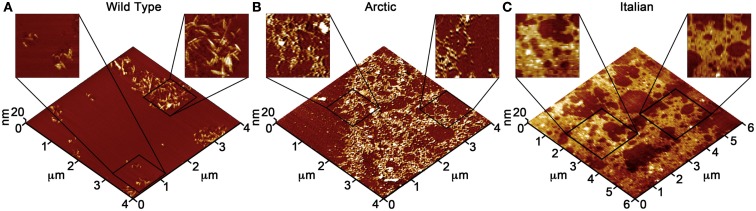
**Point mutations in Aβ influence peptide aggregation in the presence of total brain lipid bilayers**. Using solution AFM, aggregation of Wild Type, Arctic (E22G), or Italian (E22K) Aβ in the presence of supported TBLE bilayers was monitored (Aβ concentration was 20 μM for all experiments). 3D images are presented (4 μm × 4 μm and 6 μm × 6 μm) with indicated zoomed in areas of 1 μm × 1 μm and 2 μm × 2 μm shown in 2D. **(A)** With time, Wild Type Aβ aggregated into discrete oligomers and fibrils that were associated with regions of the bilayer with perturbed morphology (an increase in surface roughness). **(B)** While many small oligomers of Arctic Aβ were observed on the bilayer, highly curved fibrils that were associated with membrane disruption were the dominant aggregate species. These Arctic Aβ fibrils were morphologically distinct from fibrils observed for Wild Type Aβ. **(C)** While Italian Aβ also formed similar oligomers compared Wild Type and Arctic Aβ, large patches of disrupted bilayer morphology developed that may be associated with distinct fibril aggregates.

A potential mechanism for amyloid-forming proteins, such as Aβ, is their ability to alter membrane structure and integrity, leading to permeation of cellular membranes (Figure 4). Detergent-like effects arise from the amphiphilic nature of Aβ, leading to reduced membrane surface tension leading to membrane thinning and hole formation (Hebda and Miranker, 2009). Several AFM studies performed in solution have provided valuable insight into the aggregation of Aβ on a variety of model lipid membranes, leading to altered membrane morphology. The interaction of Aβ(1–40) with bilayers formed from total brain lipid extract (TBLE) revealed that Aβ(1–40) will partially insert into bilayers, growing into small fibers (Yip and McLaurin, 2001). In the same study, larger fiber-like structures associated with disruption of the bilayer morphology and integrity were observed as measured by increased surface roughness and formation of holes, respectively. Large fibrils were often highly branched and associated with edges of disrupted bilayer. The TBLE bilayers also aided in nucleation and enhancement of fibril growth. Interestingly, preformed fibrils were not capable of disrupting the TBLE bilayers, which may indicate that the act of aggregation, that is pre-fibrillar aggregates, may be key in Aβ-induced membrane disruption. Similar experiments exposing DMPC bilayers to Aβ(1–40) resulted in the formation of globular aggregates that were associated with small holes in the bilayer, whereas, fibril growth and/or extensive bilayer disruption was not observed. Aβ(1–42) demonstrated a different interaction/aggregation pattern on TBLE bilayers (Yip et al., 2002). Discrete molecules of Aβ(1–42) could be detected on the surface that were replaced by distinctly larger aggregates with time. However, bilayer defects were rarely detected upon exposure to Aβ(1–42). Point mutations in Aβ(1–40) also altered the aggregation on and ability to disrupt TBLE bilayers (Figure 5; Pifer et al., 2011). These same point mutations were shown to cause polymorphic aggregation of Aβ on mica. Aggregation in the presence of TBLE bilayers resulted in a variety of polymorphic aggregates in a mutation dependent manner and a variable ability to disrupt bilayer morphology/integrity. Such results highlight the potential role electrostatic and hydrophobic properties of Aβ play in its ability to bind, insert, and potentially disrupt lipid membranes.

Another proposed toxic mechanism points to Aβ‘s ability to alter cellular ion concentrations, calcium in particular, through the formation of membrane pores (Figure 4D). Initial evidence for this scenario came from the observation that PS bilayers that had Aβ(1–40) directly incorporated into them displayed linear current/voltage relationships in symmetrical solutions (Arispe et al., 1993a). Further evidence for this scenario was provided by studies on phospholipid vesicles that had either Aβ(1–42) (Rhee et al., 1998) or Aβ(1–40) (Lin et al., 1999) directly incorporated into them. In both cases, these vesicles stiffened in the presence of calcium, due to calcium ion-induced charge–charge repulsion inside the vesicles, binding of calcium to lipids and proteins, and an enhanced efficiency of lipid–protein interactions. This increased stiffness of the vesicles could be blocked by pretreatment with anti-Aβ antibodies, Tris, or zinc, all of which would block putative calcium channels. Reconstituting Aβ(1–42) with a planar lipid bilayer resulted in the formation of multimeric channel-like structures with symmetries suggesting tetramer or hexamer pore-like structures of Aβ(Lin et al., 2001; Quist et al., 2005). The formation of a variety of similar aggregate structures in lipid membranes have also been demonstrated computationally (Capone et al., 2012; Tofoleanu and Buchete, 2012).

Similar impacts on membranes due to exposure to Aβ have been detected in cellular models. Cells exposed to Aβ(1–40), Aβ(1–42), and Aβ(25–35) on endothelial cells undergo morphological changes and cell disruption, with the highest sensitivity to Aβ(1–42) (Zhu et al., 2000). While cell disruption was induced by nanomolar concentrations of Aβ(1–42), micromolar concentrations of Aβ(1–40) were required to trigger similar effects. Similar observations were reported for fibroblasts in the presence of nanomolar Aβ(1–42), as morphological changes along the periphery of the cell were observed that could be blocked by anti-Aβ antibodies, zinc, and the removal of calcium (Zhu et al., 2000). Protofibrils and low molecular weight oligomers of Aβ can alter the electrical activity of neurons and reproducibly induced toxicity in mixed brain cultures in a time- and concentration-dependent manner, suggesting changes in membrane integrity and depolarization (Hartley et al., 1999). Aβ peptides induce ion channel-like ion flux in model lipid membranes and neuronal membranes independent from the ability of Aβ to modulate intrinsic cellular ion channels or transporter proteins (Capone et al., 2009) Even intracellular forms of Aβ can alter the electrophysiological properties of cultured human primary neurons (Hou et al., 2009). Aβ(1–42) oligomers form single ion channel permeable to Ca^2+^ in oocytes are highly toxic and not attributable to endogenous oocyte channels (Demuro et al., 2011).

The discussed potential mechanism of Aβ toxicity associated with lipid membranes are not exhaustive, nor are they mutually exclusive. Aβ-induced membrane disruption of POPC, POPOC/POPS/gangliosides, and TBLE systems occurred in a two-step process (Sciacca et al., 2012). The initial step involved the formation of ion-selective pores, followed by non-specific fragmentation of the lipid membrane due to fibrillization. This demonstrates that different mechanism of membrane disruption may be associated with specific stages of aggregation. Large unilamellar lipid vesicles (LUVs) encapsulating self-quenching fluorescent dyes can be used as reporters of membrane disruption and leakage. Such systems have been used to elucidate the ability of Aβ to disrupt membrane integrity. Upon exposure of LUVs comprised of DMPC and containing calcein to Aβ(1–42) oligomers, the LUV structure is disrupted, allowing leakage of the dye, but preformed fibrils have a decreased ability to disrupt LUVs (Williams et al., 2010). However, the oligomers that interacted with the DMPC LUVs formed fibrils, suggesting that the aggregation process may actually play a role in membrane disruption.

A variety of studies indicate the ability of Aβ to bind membranes is highly dependent on the presence of specific lipid components, i.e., cholesterol (Yip et al., 2001; Reiss et al., 2004; Yu and Zheng, 2012), sphingolipids (van Echten-Deckert and Walter, 2012), gangliosides (McLaurin and Chakrabartty, 1996), and neutral or charged phospholipids (McLaurin and Chakrabartty, 1997; Sabate et al., 2005, 2012). This may be due to specific chemical/electrostatic interactions between membrane components and Aβ and/or the mechanical properties of the bilayer associated with their specific composition. For example, altering the cholesterol content of supported TBLE bilayers changes Aβ aggregation on membranes. Aβ(1–40) induced bilayer disruption and its ability to form fibrils on the bilayer was strongly dependent on cholesterol content of the supported bilayers (Yip et al., 2001). Cholesterol depletion of bilayers inhibited the ability of Aβ(1–40) to perturb bilayer structure. When Aβ(1–40) was added to TBLE bilayers that had been enriched with 10% exogenous cholesterol, discrete Aβ(1–40) peptides appeared on the bilayer within ∼30 min. Eventually, ring-like Aβ(1–40) structures with diameters of 55–80 nm as well as short fibrils and small aggregates were observed on the cholesterol enriched bilayer, but no membrane disruption was observed. At higher cholesterol content (30% of the total lipid), these Aβ(1–40) aggregates were not observed. The ability of Aβ(1–40) to disrupt the TBLE bilayers with varying amounts of cholesterol correlated with bilayer fluidity, indicating that decreased fluidity (modulated by cholesterol content) of the membrane somehow enhanced the interaction between the bilayer and Aβ. Simulation of POPC bilayers containing different mole fractions of cholesterol demonstrate that cholesterol induces changes in bilayer properties, i.e., membrane structure, dynamics, and surface chemistry, that cause increased bilayer thickness, ordering of hydrophobic chains, surface hydrophobicity, and decreased lipid mobility (Yu and Zheng, 2012). These effects promoted the binding of Aβ(1–42) to the model POPC lipid bilayers.

Cholesterol is also critical in the insertion of oligomeric forms of Aβ(1–42) into POPC membranes (Ashley et al., 2006). With DOPC model bilayers, the addition of cholesterol acts as a target for the binding of Aβ to the membrane (Drolle et al., 2012). AFM studies further illustrate that the alteration of bilayer mechanical properties induced by lipid composition impact the ability of Aβ to bind membranes, by demonstrating that astrocyte secreted lipoprotein particles containing different isoforms of apolipoprotein E (apoE), of which the apoE4 allele is a major risk factor for the development of AD, protect TBLE bilayers from Aβ(1–40) induced disruption (Legleiter et al., 2011). The apoE4 allele was less effective in protecting these bilayers from Aβ(1–40) compared with their apoE3 counterparts, and further analysis revealed that this was due to the varying ability of the lipoprotein particles containing different alleles of apoE to modulate the fluidity of bilayers by acquiring bilayer components (most likely cholesterol and/or oxidatively damaged lipids). There is evidence that peptide/membrane affinity in vascular cells can also be related to the ability of cholesterol to modulate membrane fluidity and structure (Subasinghe et al., 2003). Other cell culture assays, using PC-12 and SH-SY5Y cells, demonstrated that depleting cells of cholesterol increased the cellular binding of Aβ(1–40) (Yip et al., 2001).

While the mechanical properties of bilayers can influence their susceptibility to Aβ binding, once Aβ binds a membrane, this association may also alter the mechanical properties of the membrane, leading to dysfunction. Such a scenario is plausible considering the observed morphological changes associated with lipid membranes exposed to Aβ(Yip et al., 2002; Legleiter et al., 2011; Pifer et al., 2011). Anisotropy studies with POPC and POPG lipid membranes demonstrated that monomeric Aβ had initially little impact on bilayer fluidity; however, oligomers were able to decrease bilayer fluidity (Kremer et al., 2000). Furthermore, oligomers prepared at pH 6 had a larger impact on bilayer fluidity compared to oligomers that formed at a neutral pH, suggesting that distinct, polymorphic oligomers were formed under the different conditions (Kremer et al., 2000). Studies performed on supported phospholipid membranes revealed that exposure to Aβ modifies morphology and local mechanical properties of bilayers, reducing the force required to break through the membrane with an AFM probe (Dante et al., 2011). The lysis tension of unilamellar vesicles containing oxysterols are altered by exposure to nanomolar concentration of Aβ peptides (Kim and Frangos, 2008). Collectively, these results suggest Aβ can negatively impact the mechanical integrity of lipid membranes.

The major risk factor associated with AD is age. Age-related changes in membrane composition and/or physical properties may facilitate an increased cellular susceptibility to Aβ cytotoxicity. For example, both enhanced cellular cholesterol content (Wood et al., 2002; Cutler et al., 2004; Panchal et al., 2010) and oxidative damage (Chen and Yu, 1994; Choe et al., 1995) are associated with aging, decreased fluidity of membranes, and AD. Oxidative damage of polyunsaturated fatty acids, in general, increase lipid bilayer rigidity as a result of increased steric hindrance restricting the movement of lipid acyl chains (Choe et al., 1995; Choi and Yu, 1995). Furthermore, Aβ oligomers display preferential accumulate at oxidatively damaged plasma membranes of cells (Cecchi et al., 2007), and there is evidence of enhanced oxidative damage in AD brains (Williamson et al., 2008; Ansari and Scheff, 2010). Such studies suggest that altered membrane mechanics play a role in facilitating Aβ/lipid interactions.

## Surface Aggregation of Other Amyloid-Forming Proteins

Surfaces can also modulate the aggregation of other amyloid-forming proteins associated with neurodegenerative diseases. Specifically, these proteins may also alter membrane homeostasis, presumably via similar mechanisms as described for Aβ. Here we will briefly discuss some features of the interaction of other amyloid-forming proteins: htt, α-syn, apolipoprotein C-II, and prions. Many studies of the aggregation of these two proteins have focused on lipid vesicles or organelles, which have membranes comprised predominately of lipids, and these studies further highlight how aggregation can be modulated by membrane composition.

## The Interaction of α-syn with Surfaces

Parkinson’s disease is a neurodegenerative disease caused by the sporadic misfolding and aggregation of the protein α-synuclein (α-syn) leading to the appearance of inclusions termed Lewy bodies. Electron microscopy (EM) and *ex situ* AFM have shown that, while a heterogeneous population of oligomers, protofibrils, and annular aggregates exists (Conway et al., 2000; Apetri et al., 2006), over time the predominant aggregate species are fibrillar (Conway et al., 1998; Narhi et al., 1999; Apetri et al., 2006). While Lewy bodies have long been known to be comprised of fibrils (Duffy and Tennyson, 1965) it is now widely believed that pre-fibrillar and pre-Lewy body inclusions aggregates are responsible for disease. The fragmentation of the Golgi apparatus, for example, corresponds to the appearance of protofibrils, rather than fibrils (Gosavi et al., 2002). This notion that pre-fibrillar aggregates are the cause of disease is supported by dementia with Lewy bodies patient brains lysates containing elevated levels of α-syn oligomers (Paleologou et al., 2009) compared to control and AD patient brains. Toxicity in cell models is usually displayed without fibrillar or protofibrillar species, but rather a 54–83 kDa aggregate, perhaps comprised of 17 kDa oligomers, that appears to mediate neurotoxicity (Xu et al., 2002). Transgenic mice, unlike human patients, exhibited neurodegeneration and inclusions comprised of fine granular material and clear vacuoles, not fibrils (Masliah et al., 2000). Furthermore, appearance of Thioflavin T (ThT) reactive aggregates have been shown to correspond with decreased fluidity of lipid acyl chains in membranes (Smith et al., 2008).

Surface stabilized α-syn aggregates have been observed by in solution AFM studies, where fibrillar sheets grew in length along two directions 120° from each other reflecting the pseudo-hexagonal surface geometry of muscovite mica. Altering the surface substrate from mica, a hydrophilic surface, to highly order pyrolytic graphite, a hydrophobic surface, impeded sheet formation, demonstrating a specific surface dependent growth mechanism (Hoyer et al., 2004). This is contrary to what has been seen in EM and *ex situ* AFM studies in which α-syn is aggregated in bulk solution. In these studies, α-syn forms oligomers and fibrils without any discernible directionality (Conway et al., 2000).

In pre-synaptic termini, α-syn exists in both free and plasma membrane or vesicle bound states (McLean et al., 2000). Densitometric analysis of rat brain fractionation demonstrated that ∼15% α-syn in the supernatant is membrane bound (Lee et al., 2002). Homozygous deletions of α-syn in mouse models and overexpression of α-syn in a neuronal cell line corresponded with changes in membrane fluidity and cellular fatty acid uptake and metabolism (Sharon et al., 2003; Castagnet et al., 2005; Golovko et al., 2005). Similarly, α-syn has been shown to have a strong interaction with synthetic anionic phospholipid vesicles (Davidson et al., 1998; Jo et al., 2000; Ramakrishnan et al., 2003), crude brain vesicles, cellular membranes, lipid rafts, and lipid droplets (Jensen et al., 1998; McLean et al., 2000; Cole et al., 2002; Fortin et al., 2004). EPR studies have demonstrated that the α-syn helix extends parallel to the curved lipid (Jao et al., 2008), while electron microscopy experiments note α-syn’s ability to tubulate vesicles (Varkey et al., 2010). *Ex situ* AFM studies of PG vesicles exposed to α-syn lead to membrane fragmentation (Volles et al., 2001) and in solution AFM experiments of α-syn aggregation on mica supported lipid bilayers demonstrate that α-syn association leads to bilayer disruption and eventual fibril formation on the exposed mica surface (Jo et al., 2000). This interaction with lipid structures is believed to be directed by the first N-terminal 60 amino acids of α-syn, which contains an amphipathic α-helix structurally similar to apolipoproteins-binding domains (Clayton and George, 1998).

Thus, the first 60 amino acids of α-syn causing subcellular localization may lead to 1) an increase in local α-syn concentration and nucleation sites or 2) the α-helical structure of the membrane bound α-syn might impede misfolding into high-ordered aggregates. Supporting the first hypothesis, FTIR and far-UV CD studies demonstrate that aggregation of α-syn depends on the proximity of the membrane; amorphous aggregates were formed on or close to membranes whereas fibrillar aggregates were formed distant to membranes (Munishkina et al., 2003). Fluorescence and AFM experiments with polytetrafluroethylene balls and α-syn also highlight that aggregate formation is dominated by reactions at hydrophobic interfaces, like lipid membranes (Pronchik et al., 2010). Similarly, fluorescence studies on supported lipid bilayers demonstrate that α-syn clustering on membranes is a function of anionic lipid and/or protein concentration (Pandey et al., 2009). Double electron–electron resonance studies reveal well-defined α-syn aggregates with lipids that could form part of larger aggregates and serve as nucleation sites (Drescher et al., 2010). The second hypothesis that α-helical membrane bound α-syn impedes aggregation into higher ordered aggregates, is supported a fluorescence resonance energy transfer study, where membrane binding alters the tertiary conformation of α-syn such that oligomerization is inhibited (Narayanan and Scarlata, 2001). However, it is important to note that the two hypotheses may not be mutually exclusive. It is possible that α-syn binding to a membrane stabilizes and nucleates a toxic aggregate specie.

Circular dichroism studies have also hinted at surface altered aggregate species as α-syn in PBS is in a random coiled secondary structure, whereas α-syn in the presence of POPC/POPS small unilamellar vesicles (SUVs) formed an α-helical structure. These studies further demonstrated that α-syn aggregation was not an effect of surface curvature as POPC/POPS multilamellar vesicles (MLVs), POPC/POPI, and POPC/POPA SUVs do not result in α-syn α-helical structure, whereas with the addition of PE to POPC/PI and POPC/POPA SUVs α-syn’s α-helical content increased (Jo et al., 2000). These studies suggest that surface membrane composition plays a role in stabilizing aggregates. Stabilized annular aggregates have been found in *in vitro* studies and human brain samples. This stabilized pore-like structure is hypothesized to lead to membrane ion leakage (Lashuel et al., 2002; Pountney et al., 2004). Thus, surface stabilized aggregates, such the membrane stabilized annular aggregates, may be one key toward understanding the mechanism of toxicity in PD.

## The Interaction of Huntingtin with Surfaces

Huntington’s disease is another neurodegenerative disease caused by a polyQ expansion within exon1 htt. The length of the polyQ domain is intimately correlated to age of onset and severity of disease (Snell et al., 1993; Penney et al., 1997; Tobin and Signer, 2000). Inclusion bodies, the hallmark of disease, once thought to be the toxic species, have been shown by a survival analysis to potentially have a beneficial rather than pathogenic response to htt aggregation (Arrasate et al., 2004). AFM experiments with both GST-fusion htt exon1 proteins and synthetic polyQ peptides demonstrate a heterogeneous and complex aggregation mechanism, including oligomers, fibrils, annular aggregates, and inclusions, in which antibodies detect numerous different conformations of these aggregates (Legleiter et al., 2009, 2010). Analytical size exclusion chromatography experiments have demonstrated that flanking sequences of the polyQ domain alter aggregation rates considerably. Specifically, in bulk solution the first 17 N-terminal amino acids accelerate aggregation while the a C-terminal polyproline (polyP) domain retards aggregation rates (Thakur et al., 2009). Different types of HD models have shown that in neurons, both normal and mutant htt proteins localize to several subcellular compartments, such as endosomes, pre-synaptic, and clathrin-coated vesicles, and dendritic plasma membrane (Harjes and Wanker, 2003). Furthermore, htt inclusion bodies developed in cell lines expressing large N-terminal htt fragments incorporate multi-vesicular membranes, autophagosomes, and mitochondria into their surfaces (Kegel et al., 2000; Qin et al., 2004).

Immunohistochemical studies and subcellular fractions have also highlighted the fact the htt is enriched in membrane-containing fractions (Gutekunst et al., 1995). In fact, ∼50% of endogenous htt distributes with membranes after subcellular fractionation of neuron-like clonal striatal cells (Kegel et al., 2005). Thus, the wide subcellular localization and membrane-incorporated aggregates suggest that there is a strong htt interaction with lipid bilayers, which may be directed by the first 17 amino acids on the N-terminus of htt exon1. This domain appears to adopt a highly conserved amphipathic α-helix with membrane binding properties (Atwal et al., 2007), which may be facilitated by the polyP domain on the C-terminal side of the polyQ domain (Qin et al., 2004). Similar to PD, subcellular localization of htt may lead to a local increase in htt concentration creating aggregation nucleation sites or stabilization of the α-helical conformation may actually stabilize specific aggregate species that are transiently formed in bulk solution. It is possible that htt association to membranes nucleates some types of aggregation while potentially stabilizing specific intermediates along that aggregation pathway.

Surface stabilized aggregates of simple polyQ peptides have been observed via in solution AFM studies that demonstrated that, while the majority of peptide formed extensive fibrillar networks, discrete oligomers formed on a mica surface (Legleiter et al., 2010; Burke et al., 2011). These studies are contrary to previous assumptions based on bulk solution experiments that aggregation of pure polyQ peptides proceeded directly from monomer to fibril without oligomeric intermediates (Chen et al., 2002a,b). Similar to α-syn, htt has also been observed by CD to alter its structure in the presence of POPC and POPS:POPC SUVs, both compositions of endoplasmic reticulum (ER) and ER derived vesicles (Atwal et al., 2007). These studies were able to show that while htt does have α-helical content in free solution, α-helical content is altered in the presence of SUVs. Interestingly, although no structural data was provided, densitometry data from Western blots were able to demonstrate that htt/lipid interaction is modulated by membrane composition and polyQ length (Kegel et al., 2009). Here, increased polyQ length had a preferential association with multivalent phospholipids. Stabilized oligomers have been identified to be associated with mitochondrial structural proteins in HD patient brains. Here, it is believed that the oligomeric species lead to mitochondrial fragmentation, abnormal mitochondrial dynamics, and oxidative DNA damage (Shirendeb et al., 2011). Surface stabilized htt aggregates, such as mitochondrial stabilized oligomeric species, may lead to understanding potential toxic mechanisms and therefore therapeutic targets.

Posttranslational modifications of htt further modify its trafficking and interaction with membranous cellular surfaces. Sumoylation of the first 17 N-terminal amino acids in htt exon1 leads to its being trafficked to the nucleus (Steffan et al., 2004). This sumoylation of mutant htt also increases soluble diffuse aggregates that elicit greater cytotoxicity and neurotoxicity in HD *Drosphila* models (Steffan et al., 2004). More specifically, when Rhes, a protein selectively localized in the striatum that increases sumoylation in transgenic mice, is overexpressed in mutant htt knock-in striatal cells, cell survival is reduced by 60% whereas there is no effect with wild type htt (Subramaniam et al., 2009). Similarly, phosphomimetic mutations at serine 13 and 16 have been shown to alter the kinetics of aggregation by reducing fibrillization while accumulating alternative aggregates (Gu et al., 2009). YAC128 mouse models demonstrated that ganglioside GM1 treatment induced phosphorylation at serines 13 and 16 resulting in a restoration of normal motor behavior (Di Pardo et al., 2012). Furthermore, structural studies have determined that phosphorylation of serines 13 and 16 inhibit the first 17 N-terminal amino acids’ amphipathic α-helix, altering the localization of htt within cells (Atwal et al., 2011). Collectively, these posttranslational modifications of the N-terminal domain modulate its lipid-binding properties and the cellular trafficking of htt.

## The Interaction of apoC-II with Surfaces

Not all amyloid diseases are neurodegenerative in nature, and insights into the ability of lipid association to promote specific aggregation pathways and structure can be gleaned from these systems. One such illustrative system is amyloid deposition associated with aortic atherosclerotic lesions (Westermark et al., 1995; Mucchiano et al., 2001; Rocken et al., 2006), which contain numerous plasma apolipoproteins, such as apolipoprotein C-II (apoC-II; Medeiros et al., 2004). Lipid stabilized conformations of apoC-II have long been observed. CD studies have shown apoC-II exists in a highly disordered conformation in bulk solution (Tajima et al., 1982), whereas, in the presence of sodium dodecylsulfate, trifluoroethanol, and phosphatidylcholine vesicles apoC-II adopts a helical structure (Tajima et al., 1982). Furthermore, in the absence of lipid, TEM, and AFM studies have revealed that apoC-II forms stable fibrillar ribbons (Hatters et al., 2000; Teoh et al., 2011) with increased β-sheet content as measured by CD (Hatters et al., 2000), whereas TEM and turbidity assays have demonstrated that DHPC micelles inhibit amyloid formation while inducing α-helical formation believed to be amphipathic (Hatters et al., 2001). ThT fluorescence assays have even demonstrated that a 1:4 apoC-II_60–70_ peptide to D5PC lipid ratio is sufficient to inhibit fibril formation up to 24 h (Hung et al., 2008). In the presence of sub-micellar phospholipid concentrations, apoC-II forms a tetrameric structure that when seeded forms apoC-II fibrils, thus indicating that the tetramer specie is on-pathway to fibril formation (Ryan et al., 2008).

Intriguingly, TEM and CD studies have observed apoC-II polymorphisms by altering the lipid environment present during fibrillization. Under low-lipid concentrations, two apoC-II populations are observed in solution, which are believed to have competing fibril assembly pathways resulting in two distinct fibril structures. One fibril structure is believed to occur via the same pathway as “lipid-free” conditions, resulting in the rapid formation of ribbonlike fibrils. The second fibril structure results in a slower development of straight fibrils and is believed to form from the remaining population of lipid-associated apoC-II. Furthermore, the population of ribbonlike fibrils appears to decline as the straight fibrils are assembling, thus it is believed that apoC-II is able to transition from a mature ribbonlike fibril into the straight fibrillar assembly pathway (Griffin et al., 2008). Therefore, the lipid stabilized straight fibrils may be key toward understanding the toxic mechanism associated with atherosclerosis. The mechanisms by which lipids trigger specific aggregate forms of apoC-II may inform us concerning similar phenomena in neurodegenerative diseases.

## The Interaction of Prions with Surfaces and Parallels with Cell to Cell Transport of Amyloid-Forming Proteins

Of all of the neurodegenerative diseases, prion diseases (or transmissible spongiform encephalopathies) have long been considered unique due to their infectious nature (Prusiner and Hsiao, 1994; Prusiner, 1998; Aguzzi and Calella, 2009). Prion diseases are caused by the posttranslational misfolding of the benign, α-helical prion protein cellular isoform (PrP^C^) into an infectious disease-related, β-sheet rich form (PrP^Sc^; Caughey et al., 1991; Pan et al., 1993). AFM and EM experiments using PrP proteins and fragments demonstrate a complex aggregation mechanism involving oligomers (Serio et al., 2000), polymorphic fibrils (Anderson et al., 2006), and amorphous aggregates (Pan et al., 1993). Prions replicate by forcing PrP^C^ of the host animal to adopt the PrP^Sc^ form, and this infectious, protein-only mechanism is now widely accepted (Soto, 2011).

As exposure to the PrP^Sc^ form occurs extracellular, the interaction of prions with the exterior surface of cells may play an important role in a variety of toxic or infectious mechanisms. Several studies point to a role for lipid membranes in the conversion of PrP^C^ to PrP^Sc^ that leads to aggregation (Stahl et al., 1990; Sanghera and Pinheiro, 2002; Robinson and Pinheiro, 2010). The model prion protein fragment (PrP118-135) undergoes conformational and orientational changes in model POPG lipid bilayers (Li et al., 2012). Furthermore, the interaction between prions and cellular membranes lead directly to liposome fusion and apoptotic cell death (Pillot et al., 1997, 2000). SDS-PAGE and subcellular fractionation studies demonstrated that PrP^C^ is a glycosyl-phosphatidylinositol (GPI)-anchored cell surface protein (Oesch et al., 1985; Meyer et al., 1986), and fluorescence studies have indicated that membrane environment alters the conformation of recombinant PrP lacking a GPI anchor (Morillas et al., 1999), playing an important role in the initial formation of the PrP^Sc^ form. Furthermore, lipid rafts or caveola-like domains are believed to be involved in the conformational transition of PrP (Gorodinsky and Harris, 1995; Vey et al., 1996). FTIR studies of PrP^C^ binding to lipid membranes composed of DMPC, sphingomyelin, cerebroside, and cholesterol observed PrP^C^ forming β-sheets at the membrane interface as the concentration of PrP^C^ reached a concentration threshold (Elfrink et al., 2008). Studies using CD spectroscopy also demonstrated that β-sheet formation in PrP106-126 fragments is induced by the clustered negative surface charges on a lipid membrane surface (Miura et al., 2007). Recently, an immunofluorescence analysis of MYC-tagged PrP^Sc^ exposed to Rocky Mountain Laboratory mouse prions was able to demonstrate that the infectious isoform PrP^Sc^ was present primarily located at the plasma membrane within 1 min of exposure (Goold et al., 2011). Collectively, these studies suggest that the exterior surfaces of cell may play a role in the initial formation of PrP^Sc^ and its subsequent propagation.

Recently, several other amyloid-forming proteins have been shown to have prion-like infectious properties. The ability to circumvent the lag-phase of amyloid formation by adding preformed aggregates in a process called seeding (Jarrett and Lansbury, 1993; Lansbury, 1997; Paravastu et al., 2006; Nonaka et al., 2010; Jucker and Walker, 2011; Serem et al., 2011), demonstrating that such seeds can impose aberrant structure on other proteins. Such a phenomenon appears to play a role in the cell to cell translation of the disease state across specific regions of the brain (Vonsattel and DiFiglia, 1998; Braak et al., 2003; Ravits et al., 2007; Braak and Del Tredici, 2011), and this is reminiscent of the infectious nature of prions. Acceleration of AD has been observed in several transgenic mouse studies by the injection of preformed Aβ aggregates, suggesting that Aβ may have self-propagating conformations that can seed aggregation *in vivo* (Kane et al., 2000; Meyer-Luehmann et al., 2006; Stohr et al., 2012). A similar phenomenon has been observed for tau (Clavaguera et al., 2009) and α-syn (Mougenot et al., 2012). While cellular membranes may still represent a target in many toxic mechanisms, for predominately extracellular Aβ, the ability of misfolded conformers to induce/seed aggregation does not necessarily depend on cellular uptake. However, for seeding of protein aggregation associated in neurodegenerative diseases associated with intracellular inclusions/deposits, uptake of the self-propagating conformers is necessary, and this may be facilitated by the interaction with the cell membrane or other lipid-containing structures. Cellular uptake of aggregates of several amyloid-forming proteins has been demonstrated. Aggregates superoxide dismutase-1 associated with ALS can penetrate cells by macropinocytosis and seed further aggregation (Munch and Bertolotti, 2012). Pure polyQ and htt exon1 aggregates have both been shown to penetrate mammalian cells, inducing aggregation (Ren et al., 2009; Trevino et al., 2012). Experiments with cultured cells have demonstrated that extracellular aggregates of tau are endocytosed by cells, inducing the aggregation of intracellular tau (Frost et al., 2009; Nonaka et al., 2010; Guo and Lee, 2011), and the propagation of tau aggregates within the brain of a mouse model via a prion-like mechanism has been demonstrated (de Calignon et al., 2012). The ability of α-syn aggregates to seed intracellular aggregation in a variety of cellular systems (Danzer et al., 2009; Hansen et al., 2011) has also been demonstrated and mouse models (Mougenot et al., 2012) has been demonstrated. The interaction of amyloid-forming proteins with cellular surfaces may also stabilize aggregates with seeding capabilities. Such a scenario has been demonstrated for α-syn (Lee et al., 2002).

## Conclusion

While the aggregation of amyloid-forming proteins in bulk solution has been extensively studied, there is still much to understand at the molecular level about protein aggregation associated with surfaces. Of particular interest are lipid membrane surfaces, which cannot only mediate and influence protein aggregation, but also may be directly targeted by toxic protein aggregates. Due to the transient nature of several aggregate species and the continuing debate concerning specific toxic species, the mechanisms associated with the ability of surfaces, like lipid membranes, to potentially stabilize (Drescher et al., 2010) or promote (Martins et al., 2008) specific aggregates need to be further elucidated. Understanding these phenomenon may prove crucial in the effectiveness of therapeutic strategies based on manipulating the aggregation pathways of amyloid-forming proteins, as has been demonstrated for EGCG (Engel et al., 2012). The exact mechanisms associated with amyloid-forming proteins leading to cellular dysfunction and death have not fully been elucidated. The ability of such proteins to perturb membrane integrity via a variety of scenarios could directly lead to membrane dysfunction, disrupting organelles, or cellular homeostasis. Still, the specific aggregate species that cause membrane destabilization are not entirely clear, and it could be that the aggregation process itself occurring at lipid surfaces may play a critical role in damaging membranes. It is an intriguing possibility that induced changes in lipid membranes may represent a common toxic motif. Continued research into the mechanism of interaction between specific conformers capable of seeding aggregation with cellular membranes is needed to fully understand how amyloid propagates from cell to cell (Munch and Bertolotti, 2012). How specific changes in cellular properties, such as membrane mechanics, influence the susceptibility of specific cells to the prion-like propagation of these protein aggregates remain unclear (Cecchi et al., 2007). Here, we highlighted some specific features of amyloid aggregation at model surfaces and lipid membranes. While the studies reviewed here are not exhaustive, we hope that collectively they offer a compelling argument that such surface induced aggregation may play a role in a variety of toxic mechanisms associated with these diseases.

## Conflict of Interest Statement

The authors declare that the research was conducted in the absence of any commercial or financial relationships that could be construed as a potential conflict of interest.
